# A Framework for Online Document Verification Using Self-Sovereign Identity Technology

**DOI:** 10.3390/s22218408

**Published:** 2022-11-01

**Authors:** Abylay Satybaldy, Anushka Subedi, Mariusz Nowostawski

**Affiliations:** Department of Computer Science, NTNU, 2815 Gjovik, Norway

**Keywords:** document verification, SSI, blockchain technology, decentralization, trust, privacy

## Abstract

As the world is gradually moving towards digitization, forgery of vital digital documents has become relatively easy. Therefore, the need for efficient and secure verification and authentication practices of digital documents is also increasing. Self-sovereign identity (SSI) is a set of technologies that build on core concepts in identity management, blockchain technology, and cryptography. SSI enables entities to create fraud-proof verifiable credentials and instantly verify the authenticity of a digital credential. The online document verification solutions must deal with a myriad of issues in regard to privacy and security. Moreover, various challenging and tedious processes have made document verification overly complex and time-consuming which motivated us to conduct this research. This work presents a novel framework for online document verification based on SSI technology. The solution address the complexity and interoperability issues that are present in the current digital document verification systems. We look at a particular use case, i.e., document verification in online loan processing and evaluate how this proposed approach can make an impact on the existing system. Our solution based on SSI standards replaces the intermediary and enables trust between players in the ecosystem. The technology also holds the potential to make the system more efficient, interoperable, and privacy-preserving.

## 1. Introduction

The world is moving towards digitization and there is a growth of usage of digital documents. The COVID-19 pandemic has further accelerated the shift to digitizing services resulting in more interactions done online. There are many benefits that come with digitization, and at the same time new areas of vulnerability.

There are various aspects to evaluate when a physical document is presented in front of us. Many aspects of its authenticity can be acknowledged by looking at it. Physical documents have been around for thousands of years and we have developed reliable identification methods over time with strong security features. However, in a digitized document, there is a single image as opposed to a three-dimensional physical document, hence the level of sophistication is lower, leading to increase in surface area for attacks. Thus, although digital papers are handy to use, establishing their legitimacy can be difficult.

Forgery of vital digital documents has become relatively easy as a result of the technological revolution and easy access to inexpensive and modern equipment, making document authentication quite a tedious task [1]. The verification, however, has been more challenging than ever, especially in financial services which has a critical business activity rich in documents requiring intensive evaluation [2]. In addition, the process of examining documents is complex because there is no common methodology for validating and verifying electronic documents [3]. The solutions available so far are not universally compatible nor acceptable for all purposes [4]. Document verification in most enterprises is carried out through collaborative networks. These collaborative networks are quite expensive, and a lot of money and time have to be spent to put secure document verification in place. Every time a next-generation authentication method or new security module is introduced, the existing method needs to be updated for each organization. This can deviate organizations from their core business services while also affecting a customer’s experience. In addition, from the user perspective, each of us is obliged to share our information with each regulated business we interact with, eroding our privacy, security, and safety.

Self-sovereign identity (SSI) is a new model of identity management that builds on core concepts of decentralization, distributed ledger technology and cryptography, and holds the potential to make the existing systems more secure, efficient, interoperable, and user-centric. It is a new paradigm that enables individuals to have complete control over how their personal information and data is stored, shared and used. In essence, SSI allows individuals to manage their own digital documents and credentials. It also allows organizations to define their own business processes and workflows without having to rely on third-parties and central authorities. This makes SSI a powerful tool for organizations looking for self-sovereign solutions in the digital world.

In this work, we explore the use of SSI and its implications for the online document verification systems. We apply a rigorous design science research method to develop and evaluate an SSI-based online document verification framework incorporating existing theoretical knowledge through a literature review as well as collecting evidence and evaluation from the practitioners’ perspectives through consultation with experts. The overall contribution of this work is the proposal of an architectural design and the development of a proof-of-concept application for an online document verification system that is based on the SSI standards. By instantiating our framework in a proof of concept (PoC), we demonstrate our approach’s feasibility and evaluate its fitness to solve problems in online document verification systems. We look at a particular use case, i.e., document verification in online loan processing and evaluate how this novel approach can make an impact on the existing system.

The rest of the paper is organized as follows. In Section 2, we set the theoretical foundations for online document verification solutions and SSI. Section 3 introduces our research methodology. In Section 4, we derive design objectives for an online document verification solution. We then present the SSI-based framework (Section 5) and a PoC implementation details (Section 6), as our design artifacts. Section 7 describes the evaluation of our artifacts, summarizes practical implications, and elevates our research for a theoretical discussion by deriving design principles. In Section 8, we conclude and identify limitations and avenues for further research.

## 2. Background

This section lays down the theoretical groundwork for understanding different technologies necessary to comprehend the research presented in the paper.

### 2.1. Self-Sovereign Identity

**Figure 1:**
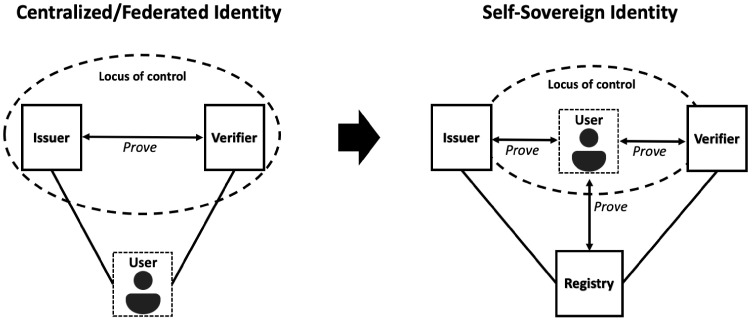
Shift of control with SSI.

SSI is a set of technologies that move the control of digital identity from third parties directly to individuals. In the centralized and federated identity models, the locus of control is with the issuers and verifiers in the network. In the decentralized SSI model, the locus of control shifts to the individual user, who can now interact with everyone else as a full peer [5]. This relation is presented in Figure 1.

SSI holds the potential to address current issues of digital identity in order to make the system secure, trustworthy, easier to use and interoperable. It does this by leveraging blockchain technology and by introducing a decentralized infrastructure to minimize trust in third parties. The sole ownership over the ability to control the user’s personal data is handed to the user in SSI. The users can then store their credentials on their devices and provide it for verification and transaction without the need to rely upon the central authority [6]. Trusted third parties thus only act as an issuer of credentials on request by the subject and cannot learn with whom or when subjects share their credentials. The SSI, in theory, thus guarantees data minimization and data control.

In addition, SSI technology is unique in a way that it serves as a digital analog for identification in the physical world. The strength of identification in the physical world is that the credential is always with the owner (such as a driving license) and is legally and practically recognized as a valid proof of identity (signature of the issuer and picture of the owner), and most importantly, it is always shared between the identity owner and the verifier without the knowledge of the issuing party.

The SSI space is growing exponentially and there are different groups and standardization agencies working to develop new standards and protocols which could be the base of the SSI model. These efforts come from agencies such as the Decentralized Identity Foundation (DIF), the European Blockchain Services Infrastructure (EBSI), the Internet Engineering Task Force (IETF), Sovrin, OASIS, the OpenID Foundation (ODIF), and the World Wide Web Consortium (W3C) [7]. To date, the two fundamental base standards for self-sovereign identities are decentralized identifiers (DIDs) [8] and verifiable credentials (VCs) [7] by the W3C. The DID and VC standards propose a common data model for unique identifiers and credentials for self-sovereign identity solutions.

### 2.2. Decentralized Identifiers

A DID is a new type of identifier that is decentralized, globally unique, resolvable, and cryptographically secure. It differs from other types of identifiers in that it can exist without the involvement of any certificate authorities, third parties, providers, or centralized identity registers.

A DID is expressed as a URI scheme; an example of a DID is “did:example:12345”. A DID is made up of three parts that are separated by colons. The "did" part of this DID represents that it is a DID, “example” is the DID method, and “12345” is the method-specific identifier that is used to distinguish this DID from other DIDs with the same method. The DID can be stored as a DID document on a blockchain or other storage system.

The DID document contains all the information required to authenticate, authorize, or interact with the subject of the DID, such as the cryptographic material and public keys. It may also contain service endpoints that describe a mechanism on how the DID subject is reached and establishes trusted communication. A DID document can be serialized in either the JSON or JSON-LD format [9]. The location of where the document is stored depends on the used DID method and may be stored either on-chain, meaning that the document is written to a blockchain, or off-chain, meaning that the document is not written to the blockchain and stored somewhere else.

The DID method describes how to resolve a DID to its associated DID document. It also specifies the operations that could be made to the document, such as how the document can be modified by the DID controller. In simple terms, a DID uses the DID method to resolve a document (DID document) that describes the subject (DID subject) to which the DID refers to and it is controlled by the DID controller. There are many different DID methods currently available, and Fdhila et al. [10] evaluated some of them, including an analysis of their qualities.

### 2.3. Verifiable Credentials

The VC data model was adopted as a standard in 2019 by the W3C. It is used to build trust between the involved parties in an SSI ecosystem, which often includes an issuer, holder, verifier, and verifiable data repository. A common procedure among the roles is that the issuer first offers the holder a VC. The credential is used by the issuer of a credential to make claims about a credential subject. A credential can hold many claims about a subject. The issuer is responsible for creating and specifying the credential’s content as well as the verification method. The verifiable credential is typically held by the credential subject, who then stores it in a digital wallet and is referred to as the holder of the credential. The credential subject can then present these claims to the verifier upon request to prove something about themselves. Lastly, the verifier then validates that the credential has not been tampered with and was issued by a trustworthy issuer, in addition to its own policy, to determine the credentials validity. The verification process can be carried out without involving the issuer directly.

A VC is made up of three main parts. First, there are the credential metadata, which consist of information that describes the credential such as credential type, who issued the credential, when it was issued, and when it expires, as well as a context property that permits an agreed-upon understanding of the credential and its structure and can be processed by JSON-LD. Second, the credential can contain statements about the credential subject in the form of one or more claims expressed as property–value pairs in the credential. Last but not least, it contains proof(s) that enable(s) the credential to be cryptographically verifiable using digital signatures. A verifiable credential can be serialized in JSON or JSON-LD, with the proof format being JWT or Linked Data.

Verifiable credentials are typically used in conjunction with decentralized identifiers to make attestations about a certain DID subject issued by a trusted DID. When presenting and validating a credential, it may be necessary to demonstrate that the holder is also the credential’s subject. Because a DID is bound to a VC via the credential subject attribute, the prover can show possession of the private key corresponding to this DID to a verifier by including verifiable credentials inside a verifiable presentation signed with this key. A device that stores verifiable credentials should also have adequate security features, such as enabling device passwords, pins, biometric data, or multifactor authentication to protect against unauthorized use.

A verifiable presentation (VP) [11] contains data that can be cryptographically verified and is commonly used to encapsulate one or more VCs. It could also include zero-knowledge proof (ZKP)-derived data and selective disclosure. In addition, the proof on the VP is often used for authenticating the holder.

### 2.4. Distributed Ledger Technology

Distributed ledger technology (DLT), often known as “blockchain”, is the technology underpinning decentralized databases that allows users to govern the generation of data across entities via a peer-to-peer network, using consensus techniques to ensure data replication among nodes [12]. SSI was born as a result of blockchain technology providing an exciting new way to establish a decentralized public key infrastructure [5]. In SSI, the blockchain acts as a replacement for the registration authority in classic identity management systems where the pairing of identification and authentication is maintained [13]. In other words, the blockchain acts as an immutable record of data used to store the public DID of the organization who issued the credential. The verifying parties can then utilize the blockchain’s infrastructure to check the authenticity of the attestation and attesting party (such as the government) from which they can determine whether to confirm the proof instead of checking the validity of the actual data in the presented evidence. An example of this could be when a holder presents a proof of their date of birth, instead of checking the accuracy of the date of birth, the verifying party will validate the government’s signature that is issued and attested to the credential. The verifier can then decide whether they trust the government’s assessment of the data’s accuracy. The first blockchain designed specifically to support SSI was created in 2016 by Evernym [14] as an open-source codebase for public permissioned ledger with all nodes controlled by trusted institutions. The codebase was subsequently contributed to the Sovrin Foundation hosted by the Linux Foundation [15], where it became Hyperledger Indy which has transaction and record types that make DID management easy. At present, the majority of SSI systems utilize the blockchain technology including Serto (Ethereum) [16], ION (Bitcoin) [17], Trinsic (Sovrin) [18], and SpruceID (Tezos, Polygon and Ethereum) [19]. From an academic research perspective, blockchain-based SSI systems are also gaining a lot of attention to introduce new solutions for digital identities [20,21,22,23,24].

Other traditional databases, such as DID registries, could also be considered for SSI. However, such databases are neither self-service nor censorship resistant. Furthermore, trust in most of these databases is based on centralized administrators whose interests may differ from those of the people they identify. In addition, when a third-party mediator knows every login or interaction, privacy is questioned. Thus, SSI is still strongly linked with blockchain technology because it requires a neutral platform that provides governance, standards, and essential public information to check the validity of attestations [25].

### 2.5. Digital Wallet and Agent

A digital wallet is software or hardware that is responsible for securely storing identity data and cryptographic content. In the context of SSI solutions, this includes storing VCs, DIDs, and the associated cryptographic keys. An SSI digital wallet should implement open standards for portable, self-sovereign VCs and other sensitive private data [5]. This means that the wallet should accept any standardized VC irrespective of the vendors and thus should have the same basic experience no matter what wallet is used. In addition, the user should be able to install the wallet on any device that they use regularly and should be able to back up and move the content to another digital wallet as required. Moreover, an SSI wallet should work with a digital agent to form connections and exchange credentials. An agent acts on behalf of the user and can communicate with other agents to do various actions; it typically accesses the digital wallet for storing and retrieving information to perform cryptographic operations. Depending on the usage, these actions can be programmed to be executed automatically by the agent or manually by the user. Furthermore, the agent can operate on an edge device or in the cloud.

## 3. Related Works

The problems related to the authenticity, reliability, and validity of an online document verification has led to various research works exploring multiple possible technologies. A number of researchers have focused on developing document verification systems based on decentralized technologies. In the literature, there are several applications based on blockchain, which make use of the benefits derived from technology as a guarantee of the authenticity, immutability, and transparency of registered information. However, little is found on how SSI technology could be utilized to solve the existing issues in document verification systems and this study seeks to fill in this gap.

In the paper by Lakmal et al. [3], a new approach (IDStack) to document verification using text extraction, digital signatures, and a correlation score for a set of documents is proposed. The IDStack protocol is implemented in a modularized architecture, which has three unique and different modules: a data extraction module, a data validating module, and a score calculating module used by three user roles called extractor, validator, and relying party. The extractor can be the owner or a third party that can validate and digitally sign the machine-readable format document. The validator can be a third-party user who can verify that the content and the previous signed machine-readable document is valid. The relying party is the one that views the document such as a bank. The protocol is accessed via the IDStack API. The proposed solution, however, involves multiple manual interactions and may be time consuming. The main problem is that all the relying parties need to have a preconnection with the IDStack and that every time a request is made by a relying party, an API is triggered in the IDStack. This is problematic in a way that it can make the user trackable, putting the user’s privacy at risk. Moreover, IDStack’s digital signing is based on self-signed certificates that allow users to create their own signatures, which raises the problem of verifying the signer’s identity.

Imam et al. [1] present a decentralized web application (DocBlock) for digital document verification using Ethereum’s blockchain. The proposed system enables a user to upload, verify, and download a digital document. A Metamask wallet is required to submit a validated document to the blockchain, with the hash of the file related to the organization’s public key and the date of the upload to further verify the authenticity of the document. If the document is corrupted at any point, the authority would know due to the change in the document’s hash value. When a document is added, the user is given an IPFS (InterPlanetary File System) hash which is given one time and the user is responsible to save it somewhere for further usage. This IPFS hash is not stored in the smart contract so the system is more private. Now, when a user enters the IPFS hash into the system, it searches for the file that matches the hash and returns the original file in binary code. Despite the fact that the paper presents a better solution than traditional technologies by enabling a transparent and auditable system, it overlooks an essential feature: user control. Once a document is uploaded to the blockchain, the user in the system may have little to no control over it.

The paper by Marella and Vijayan [26] develops a solution for the background verification process of job applicants during the hiring process using blockchain technology. The paper proposes the use of consortium blockchain where only specific issuers and verifiers have the privilege to write the information on to the blockchain. All the transactions (document submissions) should have a proper format and digital signature of the organizational entity. The system saves the hash value of the documents of the job applicants on the blockchain instead of the actual documents. However, the proposed design does not consider users and focuses on organizations (issuers and verifiers). The proof-of-concept implementation presented by authors uses Hyperledger Fabric [27], but the paper lacks the evaluation of the artifact.

Existing research into how SSI technology can be applied to document verification use cases is rather limited and underexplored. However, several researchers have studied identification and authentication methods based on DIDs and VCs. For example, Soltani et al. [28] explored SSI in the context of Electronic Know Your Customer (eKYC) where they addressed the client onboarding process and evaluated their solution on a technical level. eKYC is the process of electronically verifying the customer’s identity and credentials [29]. In the paper, they proposed a client onboarding framework named KYC2. The framework was based on Hyperledger Indy [30], a public, permissioned distributed ledger, purpose-built for decentralized identity. The framework simulated a financial client onboarding scenario among a client and two banks. The authors conducted an assessment of the framework based on the principles of SSI, General Data Protection Regulation (GDPR), and Privacy by Design (PbD). Building on the paper by Soltani et al., Schlatt et al. [25] explored the topic further extending the scope and emphasizing banks’ requirements. They built a framework to improve on the current shortcomings in the eKYC process through an end-to-end digital process that leveraged blockchain-based SSI. Their evaluation suggested that the design based on SSI significantly contributed to a more efficient eKYC process and also addressed other requirements of the stakeholders. However, the proposed framework was not implemented and lacked a feasibility evaluation. The comparison of solutions are shown in Table 1.

The SSI technology is in its relative infancy but has already been gaining traction in recent years. It has been explored for various use cases including the security of Internet of things (IoT) devices [31], biometric authentication [32], supply chain visibility [33], international payment solutions [34], and digital driving license verification [35]. It was found that SSI technology had the potential to address issues that are universal and applicable across industries.

There are a number of start-ups and companies that directly work with SSI technology to tackle the problem of digital identity management. Examples include Sovrin [15], uPort [36], Evernym [14], Trinsic [18], Mattr [37], and Civic [38].

## 4. Methodology

We followed a design science research (DSR) approach to conceptualize and evaluate a novel SSI-based online document verification framework and to derive generalizable design knowledge in the form of design principles. DSR is a research methodology that focuses on solving problems by developing artifacts through a build-and-evaluate process [39,40,41]. The build process includes all activities to create something innovative, while the evaluation aims to get feedback and to better understand the problem at hand, allowing for the artifact’s improvement [42].

**Figure 2:**
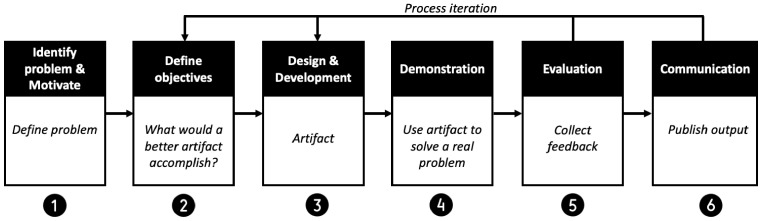
Design science research methodology.

We structured our research by following the frequently used and widely accepted DSR process of Peffers et al. [39] as illustrated in Figure 2. Since we followed a problem-centered approach, we first became aware of the problem at hand (1). Our examination of the literature and existing systems revealed several challenges, including the inefficiencies in the verification process, fraud activities, a lack of user control and privacy, and challenges in verifying the authenticity and integrity of digital documents. Based on the acquired understanding of the problem and existing requirements for the online document verification system, we then derived design objectives to overcome the identified challenges (2). This approach had six main objectives which served as a basis for creating and evaluating our artifact. In the next step (3), we designed our SSI-based document verification framework. We selected an online loan application as a use case and instantiated a proof of concept to demonstrate the usage of the artifact for solving real problems (4). We then presented the artifact to experts to get their feedback and to iteratively improve our artifact (5).

### 4.1. Use Case

A use case is presented in order to better understand the proposed framework and implement the PoC. For this, a common scenario for banking customers was employed, which was an online loan application and document verification. Based on the literature, we assumed that in order to apply for a loan, first, a user has to prove that they have performed KYC verification and can authenticate with a trustable identity provider; second, they have to prove they are employed and have a stable minimum income; and third, the user should present a tax settlement certificate issued by a tax administration from the previous year. The user needs to submit these documents to the bank and also verify the authenticity of these documents in order to be granted loans. When a user submits the required documents online, the bank should be able to fully trust the authenticity of the document presented online and this process should be straightforward so the loan can be processed as soon as possible.

### 4.2. Design Objectives

As discussed in Section 1 and Section 3, the existing method of online document verification is a lengthy process with a lot of human interpretation and third-party observations causing high possibilities of error. Moreover, the existing technology lags behind in terms of user-privacy, interoperability, and reliability. The main objective behind this work was thus to build a novel innovative solution to address the existing challenges. Based on the literature, we identified six main objectives for the new framework for digital document verification.

- **Objective 1: Efficiency**
  There is a long time involved in carrying out document verification and it is especially more challenging if it is cross-border. From the user’s standpoint, organizations that provide quick and easy document verification are more attractive. This also provides companies with a competitive advantage. Furthermore, there are several procedures involving data validation, such as determining if the document’s validity has expired, which are performed manually in many existing systems and create longer waiting times for users [43]. Thus, the automation of this manual process is also an important requirement.
- **Objective 2: Decentralization**
  Customer data silos are honeypots for hackers, thus securing valuable information is costly and not the core business of financial institutions such as banks. Similarly, any error can have serious ramifications in terms of reputation, sanctions, or both, as is also discussed in [25]. Multiple revelations of data breaches in businesses, particularly financial institutions [44,45], have resulted in major ramifications for the organization’s brand, consumer confidence in the architecture, societal trust, and personal safety. In general, consumers have lost faith in central authorities in recent years as a result of situations in which large corporations purposefully abused user data for financial or political gain [46]. This is regulated to some extent in financial institutions, but we cannot be fully confident that there is no chance of this happening in the future. As a result, data decentralization was chosen as another need.
- **Objective 3: Privacy**
  Users should be able to share their documents and credentials in a privacy-preserving way by minimizing the amount of personal information that needs to be disclosed. For example, in the case of loan processing, one may only have to prove that their salary is beyond a certain number but not an exact value. It is also important that the user has control over their documents, data, and its flow. In addition, the verification of the document must not involve contacting the issuer, because the issuer of the certificate would otherwise know where the user has been and may be able to track them. Data protection regulations (e.g., the GDPR) provide strict legal rules on storing and processing personal data. It should not be stored on the blockchain and records need to be deleted at users’ request [23].
- **Objective 4: Trust**
  Authenticity and integrity are important aspects of document verification and the documents that are submitted must be trusted by the verifiers. The digital document should not have been tampered with after being issued and it should be feasible to verify and validate it. There must exist a complete trust between different actors in the system and the user should be able to convince the verifier that their document is not stolen, sold, or shared. The submitted documents must have a higher level of assurance in a completely digital setting.
- **Objective 5: Interoperability**
  It is important that the system is based on open standards and relies on open data structures and technologies. The proposed document verification solution should be applicable across different institutions, organizations, and borders. In addition, it is also important that it can be integrated into existing systems.
- **Objective 6: Transparency**
  Transparency means that the systems and algorithms must be transparent [5]. It enables us to make an informed decision and improves accountability [47]. Both in terms of how they function and how they are controlled and updated, it is important that the technologies used to administer and operate document verification systems be easily accessible. The architecture should be open, well-known, and independent allowing anyone to examine how different components operate.

## 5. System Architecture and Design

To comprehensively address the challenges of online document verification, and to meet all the design objectives, an architecture based on SSI standards is proposed in this paper.

### 5.1. Architecture Overview

**Figure 3:**
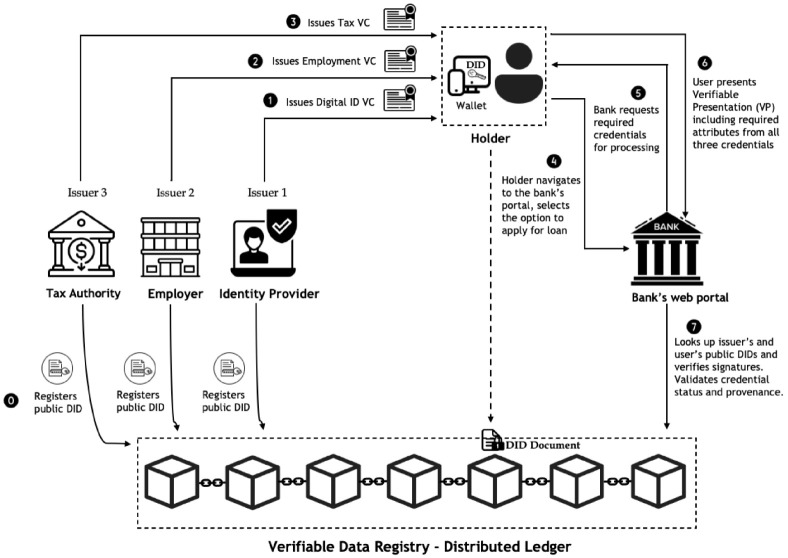
Architecture of the proposed system.

The overall architecture of the proposed system is illustrated in Figure 3. The basis of this new architecture is VCs, DIDs and the distributed ledger technology which were discussed in detail in Section 2. The architecture consists of issuers, the holder, and the verifier as actors required for document verification in online loan applications. In this context, an identity provider, employer company, and tax authority act as the issuers of VCs, the loan applicants as credential holders, and banks as verifiers.

The step-by-step working of the interplay between the actors involved in the process is given below:

- The holder applies for a VC from a trusted identity provider (IdP), which allows them to authenticate themselves to the bank’s web portal. The bank itself could act as an IdP and issue a corresponding VC to the user. The holder also needs employment and tax certificates which are issued in the form of VCs by their employer and the tax authority, respectively. The VC contains a set of tamper-evident claims about a user and metadata that cryptographically prove who issued it. When an organization issues a VC, they attach their public DID to that credential. That same public DID is also stored on the blockchain. When someone wants to verify the authenticity of the credential, they can check the public DID on the blockchain to see who issued it without having to contact the issuing party. The blockchain acts as a verifiable data registry, a “phonebook” that anyone can consult to verify what organization a specific public DID belongs to.
- The holder consents to accept/decline the issued credential. If the user accepts, they can now receive this credential and store it in their wallet which could be a web wallet or a mobile wallet. The holder stores the private key for the DID in their digital wallet, while the public keys are registered to the blockchain by the issuing authority.
- The holder/user now visits the website of the bank, authenticates with a digital ID VC stored in a wallet, and finds the relevant page for the online loan application. The bank defines the list of required documents to process the loan and sends a proof of request to the holder. The holder receives the credential share request from the bank, reviews the proof request, and determines whether the relevant credential and/or claims exist in the wallet. If the holder has all of the qualifying credentials, they can make a verifiable presentation (VP) that includes all of the required credentials. As also discussed in Section 2.3, a VP is a packaging mechanism to cryptographically prove that the holder is sending the VCs. This way the user does not have to send each credential separately but all in one transaction.
- The bank then looks up public key material from the distributed ledger and verifies signatures. The bank is capable of verifying all the electronic information (i.e., the validity of the credential, status, issuer, presenter, and claims) against the blockchain network without having to contact the original issuer. The status of a VC is checked by the verifier to ensure that it is still valid. From an implementation standpoint, W3C’s specification has a property called the credential Status property that contains information about the current status of a VC.

### 5.2. Enabling Technologies

SSI technologies are very novel, and many of the foundational standards and frameworks are still in their relative infancy. The reason behind the selection of the technologies, tools, and frameworks used in the proposed architecture are described in this section.

#### 5.2.1. SSI Standards

DIDs are cryptographically verifiable and decentralized, which are the two strong characteristics that distinguish them from other identifiers. It promotes the idea of using anonymous or pseudonymous identifiers, which makes it difficult to correlate user activities in different services thus preserving users’ privacy. Further, their decentralized nature makes credentials always available for verification. These features aligned well with the design objectives set for the creation of a trusted document verification system and thus was chosen as one of the technologies.

A VC on the other hand, is based on a standard called JSON-LD [9], which is a JSON-based format used to serialize Linked Data. It is one of the most widely used structured data formats and is a standard-based machine interpretable data across various documents and websites. It can be easily integrated into deployed systems using JSON, which is a lightweight data-interchange and already widespread as the de facto format to send data back and forth in HTTP requests and responses. In addition, features such as the selective disclosure and the support of zero-knowledge proofs give users the flexibility to show only the subset of the attributes in the credential, preserving the privacy of the user.

Traditional standards, such as X.509 certificates [48], might theoretically serve as DID documents in the SSI architecture. However, X.509 certificates are typically stored in specific servers or computers making them challenging to be portable and may create vendor lock-in. In addition, the subject’s personal information is reflected in the certificate, thus pseudonymity would not be allowed. Thus, they cannot meet the baseline criteria for interoperable, reliable, and guaranteed data privacy [49].

One more reason that DIDs and VCs were selected as technologies is because they are based on open standards developed by the W3C and have also been accepted and adopted by governments, organizations, and businesses worldwide implementing self-sovereign identity ecosystems. Open standards enable systems designed by diverse technology providers to communicate with one another. This gives a service user the flexibility to change to any provider without losing data or information [50]. In other words, the technology based on open standard alleviates concerns resulting from obsolescence or dependency issues [51]. Furthermore, the W3C is a reputable organization, working actively to develop protocols and guidelines for the standardization of web technologies. According to its website, the W3C follows processes that promote the development of high-quality standards based on the consensus of the community. Since it also reflects the views of global stakeholders while being downloadable at no cost, it is trusted and accessible to everyone to explore and examine its fundamentals [7]. As DIDs and VCs are based on open standards set by a reputed organization, they reap the benefits provided by open standards while being trusted and free of cost.

#### 5.2.2. SSI Frameworks

Although SSI technology is still in the early development phase, there are many open-source frameworks that offer tools to build the PoC. For selecting a suitable framework for our use case, SSI technology providers were collated from a comprehensive search on the internet and consultation with the experts in the field of SSI. A large number of publicly available frameworks were gathered and key desirable qualities were researched. Tools offered by these platforms were then assessed using suitable assessment methods. Some of these criteria were pointed out in the literature [52,53], while other criteria were chosen based on the specific requirement for the demonstration of the PoC. For example, it was important that the framework follow the verifiable presentation standard and provide open APIs for the implementation.

Publicly available information through websites and documentations of the individual solutions were reviewed as the first step. The brief summary of these frameworks is described below and a comparison between these frameworks is illustrated in Table 2. The table, however, serves as an illustration rather than a full list, as the work was not intended to cover all existing solutions on the market, but rather to provide a general overview of prominent and key platforms accessible in the SSI field.

- **Mattr** is an SSI platform which is actively engaged in the SSI standards and open-source community across a range of different forums [37]. The framework offers a comprehensive set of APIs and tools that take care of all the logic and could be used to manage DIDs, private keys, VCs, and VPs. Mattr was found to have well-documented APIs and tutorials. However, when we tried to reach for developer support through the given contacts on the website, we did not receive an active support.
- **Trinsic** is another player in the SSI space which provides a set of lightweight APIs for sending verifiable data. According to their website, they are tech-agnostic and based on open standards [18]. It was found that Trinsic has an active developer support and an open API as was also reported by Lalchandani et al. [53]. It has a comparatively large community and support with over 1000 developers and multiple organizations. It also has an active community on Slack and a chatbot online that offers active support for developers and anyone interested in understanding the SSI technology better. Trinsic has reduced the complexity of open-source technologies (Aries Framework .NET and Hyperledger Indy [54]) by building on top of them and exposing APIs. On the other hand, relying on Trinsic limits the potential that open frameworks could offer and provides less flexibility when implementing our PoC.
- **Affinidi** is one of the leading players in the SSI space working to enable the creation and sharing of trusted digital identities and credentials [55]. They are open-source and released under the Apache license. The Affinidi APIs were among the most well-documented, comprehensive, and purpose-built APIs available. Affinidi has an active discord server with over 400 members. Its infrastructure consists of SidetreeJS [17] integrated into the DID registry with IPFS as the content-addressable storage. In comparison to other DID approaches, Sidetree has some distinct advantages, such as a low cost, high throughput, and the built-in portability of the identifier [56]. Furthermore, MongoDB serves as the long-term cache and the Ethereum blockchain via ChainStack serves as the ledger address. The team at Affinidi is highly involved in working groups for SSI standards.
- **Veramo** is a JavaScript framework that allows developers to create and manage decentralized identifiers and verifiable credentials [36]. It claims to work closely with the W3C and DIF in order to build compatibility across different projects and initiatives in the SSI space. Similar to Mattr and Trinsic, the Veramo agent exposes all of its functionalities via a REST API with OpenAPI documentation. All functions can also be accessed on unsupported platforms via HTTP queries. The CLI implementation could be used as a guide but the API implementation has a very thorough documentation hard to follow for developers. Thus, although Veramo bootstraps the process of issuing verifiable credentials with minimal configuration while running on multiple platforms, it is not very developer-friendly from the authors’ personal experience and also backed by [57].

Based on a comprehensive review of the aforementioned frameworks, Affinidi was chosen as the framework for the development of the PoC. Although several frameworks met the requirements, Affinidi was chosen mostly because of the immediate developer support received during the initial phase. Since the technology was relatively new and so were the frameworks, it was important that the support could be instantly received and it was indeed required, notably when generating custom schemas for the use case. Affinidi provides schemas for some of the common use cases which are available on Schema Manager portal [58] but the documentation lacked information on working with custom schemas. The request body for custom schemas required extra parameters to receive a valid response which was not mentioned in their documentation anywhere. However, this was resolved smoothly as Affinidi had a dedicated support team who was available for instant communication.

Affinidi’s framework supports the did:elem method, which is based on the Sidetree protocol and resolves DIDs. Since Sidetree is a protocol for creating scalable DID networks that can run atop any existing decentralized anchoring system, it has some distinct advantages, such as a low cost, high throughput, and a built-in portability of the identifier [56], which were important for our use case. In addition, the available demo application made the on-boarding easy and had a good overview of the workflow of an SSI application.

#### 5.2.3. Web Platform

The web platform was designed using React [59], which is a JavaScript-based library for building user interfaces. In addition to the authors’ prior experience working with JavaScript and React libraries, it was also easier to get started due to the availability of a demo app (powered by Affinidi SDK) using the same front-end technology. React takes a lot of the heavy lifting out of the coding process making code simple to read and understand [60]. It is flexible and allows developers to declaratively describe user interfaces and thus was chosen as the front-end tool for the web platform.

## 6. Implementation

In this section, we describe our approach to implement an online document verification system based on the proposed architecture. Although our implementation is still a work-in-progress, our proof-of-concept prototype shows the feasibility of our design approach and allowed us to collect a preliminary insight on the efficiency of application.

The DSR methodology we followed has a Demonstration phase which involves finding a suitable real use case and using the artifact to solve identified problems. Considering this, we narrowed down our use case and focused on an online loan application and document verification in Norway. Existing online loan processing and document verification in Norway requires authentication with BankID [61]. BankID is an electronic identification system in Norway and is trusted by most of the general public. Once a user has signed in with BankID, they are asked to submit different documents in order to process the loan application. The documents usually include an employment certificate and tax assessment documents in a pdf format which need to be uploaded to the bank’s portal. The user receives an answer when the application is processed that may take a few days to multiple weeks [62]. We aimed to improve the current system and develop a novel SSI-based online document verification system. In the proposed system, a user had to prove that they had a BankID VC from Signicat [63], the tax certificate VC from the Norwegian Tax Administration (NTA) [64], and the employment certificate VC from the Norwegian University of Science and Technology (NTNU) [65]. In the next section, we describe the design process for the VC schemas based on these certificates.

### 6.1. Designing Credential Schema

According to Sovrin Glossary [15], a schema is “a machine-readable definition of a data structure”. Schemas are used to define attributes used in one or more credential definitions. Schemas are defined using different attributes depending on use cases. It was found that there were no organizations in the SSI world that defined how a credential definition or schema for a particular use case should look like. It was good in a way because standard bodies create a huge bureaucracy that stifles growth, and it would have taken an eternity to be established [66]. According to Evernym [67], the credential schemas would instead evolve organically and most likely be established through partnership between a few forward-thinking organizations, who would agree on a credential definition and standards for what is required. One of the examples of this could be Credit Union Ledger, which, although started in the United States, is now global and is contributing to establishing a global digital credential or basically a trust framework where credit institutions can come in and design together as a consortium. A similar process can be expected for credential schemas in SSI. At present, verifiable credential context and schema definitions are hypothetical, experimental, and under development but are claimed to have the potential to handle changes in the schema level efficiently. For this work, three schemas for three different credentials were required including schemas for the BankID, an employment certificate, and a tax certificate. The process to design these credential schemas is described below.

- **BankID schema**: Based on the feedback and discussion with experts from Signicat, the schema was designed according to an OpenID Connect (OIDC) (Listing 1) response that is received when a BankID verification is requested through Signicat, which is the leading provider of Norwegian BankID in Norway. Since one of the objectives of our proposed system was to be able to integrate with current systems and coexist until the SSI system is fully developed, this option looked the best for our use case.

Listing 1: OIDC response after BankID Verification (Source [61]).

 {

    "sub": "6ofVBM_uxebykmPnAYo3ORHGGYhFXRae",

    "signicat.certificate_not_after": "2022-06-27T22:59:59.000Z",

    "signicat.certificate_issuer_dn": "TestBank",

     "signicat.certificate_unique_id": "9578-6000-4-361384",

    "signicat.certificate_not_before": "2022-06-12T23:00:00.000Z",

    "name": "John Doe",

    "signicat.national_id": "199002171234",

    "birth_date": "1990-02-17"

    "given_name": "John",

    "family_name": "Doe"

    "country": "NO",

}

The BankID VC schema (Listing 2) was created based on the Norwegian BankID attributes received from the OIDC response [61]. The schema followed the VC data model and JSON-LD data syntax. Two required properties of JSON-LD that consistently showed up in the VC, namely @context and type, were defined. Contexts mapped terms that were used in VCs and VPs to URIs that explained what those terms meant in that context while the type property expressed what kind of information was in the document. Moreover, several other parameters were added that were required for decentralized verification. This included the DIDs for the holder and issuer, and the information about the status of the credential. The W3C spec [7] references the status property in Section 4.9 which states that using the credential Status property, the current status of a verifiable credential can be discovered, such as if it has been suspended or revoked. It is populated with a status API which can be accessed by verifiers to check the status of the credential. The final schema designed for the BankID VC can be seen below.

Listing 2: BankID Schema.

 {

  "@context": [

    "https://www.w3.org/2018/credentials/v1",

    "https://schema.affinidi.com/NorwegianBankIDV1V1-0.jsonld"

  ],

  "id": "claimId:cba42389c33e5a45",

  "type": [

    "VerifiableCredential",

    "NorwegianBankIDV1"

  ],

  "holder": {

    "id": "did:elem:EiAMDI8BotTYFl4DNw-x_xNQ4H8XpEN2LaWAcv0MmNASJA"

  },

  "issuer": "https://www.signicat.com",

  "issuanceDate": "2022-06-12T23:00:00.000Z"

  "credentialSubject": {

    "data": {

      "@type": [

        "Person",

      ],

      "birthDate": {

          "@type": "Date",


          "date": "1990-02-17",

      }

      "name": {

        "@type": "Name",

        "name": "John Doe",

        "givenName": "John",

        "familyName": "Doe"

      }

      "nationalID": "199002171234"

  },

  "certificate": {

    "certificateIssuer": "TestBank",

    "certificateUniqueID": "9578-6000-4-361384",

    "certificateExpiryDate": ""2022-06-27T22:59:59.000Z"",

    "country": "NO"

  },

  "credentialStatus": {

    "id": "https://www.signicat.com/status/100,

    "type": "CredentialStatusList2017"

  },

  "credentialSchema": {

    "id": "https://schema.affinidi.com/NorwegianBankIDV1V1-0.json",

    "type": "JsonSchemaValidator2018"

  },

  "proof": {

    "type": "RsaSignature2018",

    "created": "2022-06-15T14:21:10Z",

    "proofPurpose": "assertionMethod",

    "verificationMethod": "https://signicat.com/issuers/144223#key-1",

    "jws": "eyJhbGciOiJSUzI1NiIsImI2NCI6ZmFsc2UsImNyaXQiOlsiYjY0Il19..kTCYt5

      XsITJX1CxPCT8yAV-TVIw5WEuts01mq-pQy7UJiN5mgREEMGlv50aqzpqh4Qq_PbChOMqs

      LfRoPsnsgxD-WUcX16dUOqV0G_zS245-kronKb78cPktb3rk-BuQy72IFLN25DYuNzVBAh

      4vGHSrQyHUGlcTwLtjPAnKb78"

  }

}

- **Employment schema**: Affinidi provides an online portal that contains schemas applicable to a variety of use cases. It already offers a certificate of employment schema (Listing 3) that serves a similar purpose as in our proposed system. Thus, the schema defined in the Affinidi portal was reused instead of creating it ourselves so that the implementation based on this schema would be interoperable with other systems. The schema manager can be accessed here: https://ui.schema.affinidi.com/schemas. The schema used for the employment certificate from NTNU is shown below.

Listing 3: Employment Schema.
 {

  "@context": [

    "https://www.w3.org/2018/credentials/v1",

    "https://schema.affinidi.com/EmploymentCredentialPersonV1V1-0.jsonld"

  ],

  "id": "claimId:fbd51289d18e5e29",

  "type": [

    "VerifiableCredential",

    "EmploymentCredentialPersonV1"

  ],

  "holder": {

    "id": "did:elem:EiBAYArpk_r7L8EzR1YqKe4tK0n9S6_VxpT-FLOymmx-AQ"

  },

  "issuer": "https://www.ntnu.no/",

  "issuanceDate": "2022-06-23T09:04:04.059Z"

  "credentialSubject": {

    "data": {

      "@type": [

        "Person",

        "EmploymentPerson"

      ],

      "worksFor": {

          "@type": [

            "Organization",

            "Education"

          ],

          "name": "Norwegian Institute of Science and Technology"

        },

        "reference": {

          "@type": "ContactPoint",

          "name": "Alex Jorgen",

          "email": "alex.jorgen@no"

        },

        "skills": [

          "Administrative work",

          "Human resource manager"

        ],

        "offerLetter": "https://dropbox.com/offerLetter",

        "experienceLetter": "https://dropbox.com/experienceLetter",

        "salary": {

          "@type": [

            "Salary"

          ],

          "gross": {

            "@type": "MonetaryAmount",

            "value": 518000,

            "currency": "NOK"

          },

          "net": {

            "@type": "MonetaryAmount",

            "value": 30000,

            "currency": "NOK"

          },

          "frequency": "Monthly"

        }

      },

      "name": "John Doe"

    }

  },

  "credentialStatus": {

    "id": "https://ntnu.no/status/24,

    "type": "CredentialStatusList2017"

  },

  "credentialSchema": {

    "id": "https://schema.affinidi.com/EmploymentCredentialPersonV1V1-0.json",

    "type": "JsonSchemaValidator2018"

  },

  "proof": {

    "type": "RsaSignature2018",

    "created": "2022-06-28T11:29:10Z",

    "proofPurpose": "assertionMethod",

    "verificationMethod": "https://ntnu.no/issuers/565049#key-1",

    "jws": "eyJhbGciOiJSUzI1NiIsImI2NCI6ZmFsc2UsImNyaXQiOlsiYjY0Il19..kTCYt5

      XsITJX1CxPCT8yAV-TVIw5WEuts01mq-pQy7UJiN5mgREEMGlv50aqzpqh4Qq_PbChOMqs

      LfRoPsnsgxD-WUcX16dUOqV0G_zS245-kronKb78cPktb3rk-BuQy72IFLN25DYuNzVBAh

      4vGHSrQyHUGlcTwLtjPAnKb78"

  }

}

- **Tax schema**: An existing schema for a verifiable credential serving a similar purpose could not be found online so the tax assessment document offered by the NTA [68] was used as a reference to create a new tax VC schema (Listing 4). NTA provides the individual with a tax assessment document that includes various attributes regarding personal finances over the last year. The relevant attributes were selected and the VC schema for the tax certificate was created.

Listing 4: Distance constraint algorithm.
 {

  "@context": [

    "https://www.w3.org/2018/credentials/v1",

    "https://schema.affinidi.com/TaxCertificateNorwayV1-1.jsonld"

  ],

  "id": "claimId:ae6164eda5b8a10a",

  "type": [

    "VerifiableCredential",

    "TaxCertificateNorway"

  ],

  "holder": {

    "id": "did:elem:EiBwTbgxIYnXt8H6XuiVHig_w_hNL2vynZcOLUHuOaQVzw"

  },

  "issuer": "https://www.skatteetaten.no/",

  "issuanceDate": "2022-06-28T06:02:10.639Z",

  "credentialSubject": {

    "data": {

      "personalIncomeAndNetIncome": {

        "salariesAndPayments": "518000",

        "interestOnBankDeposits": "1000",

        "totalIncome": "551000"

      },

      "deductions": {

        "minimumDeductionFromOwnIncome": "106750",

        "premiumForPensionScheme": "10263",

        "totalDeductions": "117013"

      },

      "totalBasisForIncomeTax": "402000",

      "wealth": {

        "assets": "100000",

        "bankDeposits": "152000",

        "grossCapital": "252000",

        "netWealth": "252000"

      },

      "name": "John Doe",

      "year": "2021",

      "nationalID": "199002171234",

      "settlement": {

        "withholdingTax": "134800",

        "additionalAdvance": "2500",

      }

    }

  },

  "credentialStatus": {

    "id": "https://www.skatteetaten.no//status/11,

    "type": "CredentialStatusList2017"

  },

  "credentialSchema": {

    "id": "https://schema.affinidi.com/TaxCertificateNorwayV1-1.json",

    "type": "JsonSchemaValidator2018"

  },

 "proof": {

    "type": "RsaSignature2018",

    "created": "2022-07-18T21:19:10Z",

    "proofPurpose": "assertionMethod",

    "verificationMethod": "https://www.skatteetaten.no/issuers/565049#key-1",

    "jws": "eyJhbGciOiJSUzI1NiIsImI2NCI6ZmFsc2UsImNyaXQiOlsiYjY0Il19..TCYt5X

      sITJX1CxPCT8yAV-TVkIEq_PbChOMqsLfRoPsnsgw5WEuts01mq-pQy7UJiN5mgRxD-WUc

      X16dUEMGlv50aqzpqh4Qktb3rk-BuQy72IFLOqV0G_zS245-kronKb78cPN25DGlcTwLtj

      PAYuNzVBAh4vGHSrQyHUdBBPM"

  }

}


Overall, based on the feedback from experts our main objective was to reuse the existing schemas to make the system interoperable with other systems. In case there was no existing schema that fulfilled the requirements of our system, we created a new VC schema based on the attributes of real certificates. It could be challenging to design a schema and according to conversation with Marijana, an expert from Evernym [14], the best practice is always to create small credentials that serve specific purposes. It is however important to design the architecture in a way where any coloration/information and conditional things can be overlaid on top of it. The Affinidi’s Schema Manager was a great tool to create VC schemas and was aligned with our requirements. It allows to fork existing schemas or create a new schema and publish it as a searchable schema so others can reuse them. At present, schemas are being defined by issuers of credentials; however, as governance/trust frameworks become more prevalent, there is a possibility that those organizations will outline schemas for various ecosystems.

### 6.2. PoC Design and Development

The implementation followed the proposed architecture in Section 5. The PoC consists of a holder wallet, an issuer credential portal, and a verifier portal.

#### 6.2.1. Issuer

**Figure 4:**
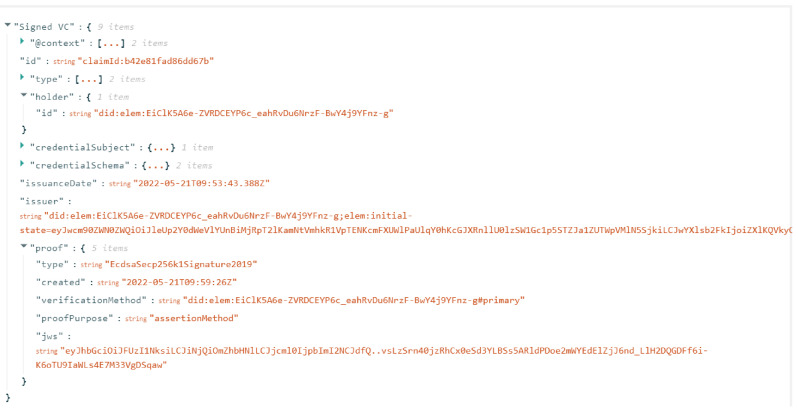
Demonstration of signed verifiable credential with cryptographic proofs.

The process starts by issuing credentials to the holder. For the PoC, all three issuers create credentials in the same web portal, but in a real-world scenario, they would each issue their own credential to the user from their own respective portal. The issuer has to first login to their portal and then fills up the necessary attributes about the holder/subject required in the credential, selects the pairwise DID controlled by the holder, and issues an unsigned credential from their defined schema. This step would be followed for all three credentials by their respective issuer, which would have their own data schema as discussed in Section 6.1. The created VC is an unsigned credential that can be used to verify if all the details in the credential are correct and there are no typos or issues with the entered data. The content of VC is checked for all possible mistakes, and it would then be signed by the issuer. Signing here refers to attaching a cryptographic proof to the credential and registering this proof in the ledger. When the process is successfully completed, the portal would show the newly issued signed verifiable credential as shown in Figure 4.

#### 6.2.2. Holder

**Figure 5:**
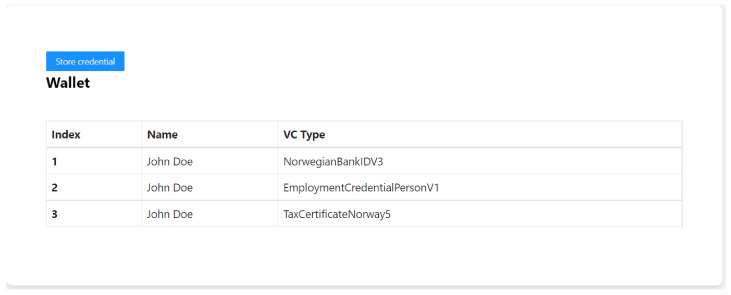
Holder’s web wallet and credentials.

In the PoC demonstration here, the holder stores the credential in a web wallet. When a user installs the wallet for the first time, they create a new account and save their wallet recovery phrase. The wallet can create an encrypted communication channel to exchange information between a user and a third party (issuer, verifier, or another user). This communication channel is based on a unique identifier, which the user controls. Users receive connection invitations which are usually transmitted through QR codes or deep links. The connection is added to the user’s wallet if they accept the invitation. When the issuer offers a credential to the user, they open the credential offer and inspect the attributes. If everything looks correct on the user end, they click the “Accept” button. The credential is issued to the user’s wallet and can be viewed in the Wallet tab as shown in Figure 5. In order to add a new credential, one can click on the “Store credential” button. This would redirect the user to a page where the user can store the new VC.

#### 6.2.3. Verifier

**Figure 6:**
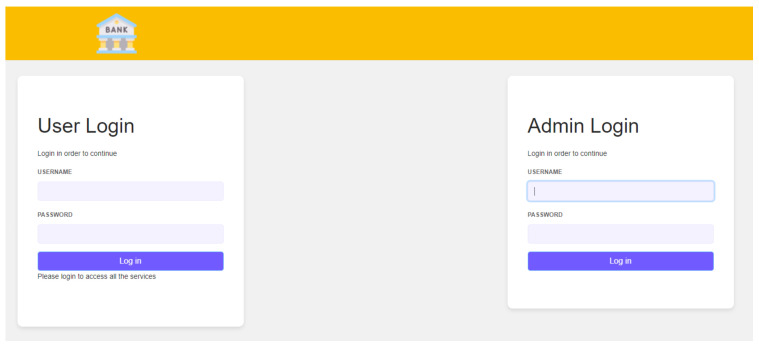
Bank portal for client and admin.

The verifier portal consists of two parts, one is the admin portal and the other is the client portal as shown in Figure 6.

**Figure 7:**
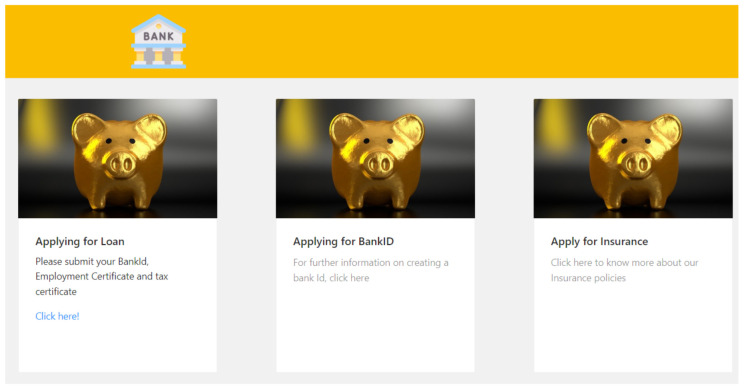
Bank’s web page with different features.

The admin portal can be used to set the credential criteria for different financial services. For example, an admin can select credentials such as BankID or tax and employment certificates as the credential requirement for processing the loan. When the holder visits the bank to apply for a loan, they log into the client side of the verifier portal. The holder then has to select the service that they are looking for. The landing page consists of different features that a user can select and in this use case, the user selects “Apply for Loan” feature as shown in Figure 7.

**Figure 8:**
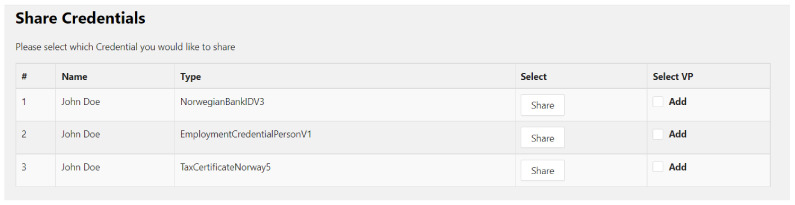
Holder’s wallet showing required credentials.

When a user clicks on the service, they receive a shared request token, consisting of the various types of documents required to take the service, and are redirected to the holder’s wallet. Here, the token is decoded to find the required document types and only those that match the types are shown on the screen (Figure 8). The implementation also supports the selective disclosure feature so that users can share the required attributes only for each verifiable credential.

When a user submits the required credentials, a share response token is created. This token is the response to the request token provided by the bank, i.e., it contains all the credentials that the bank requires to process the loan application in the form of token. The token can now be shared to the verifier through any secure medium. The verifier then has to verify if the credential token is valid. It does this by accessing the public keys and the signatures of the issuers from the VC and verifying them through the blockchain. If the credential or the token has not been tampered or revoked, it is verified successfully otherwise the verification fails.

The code for the whole implementation can be found on GitHub as a public repository maintained by one of the authors of the paper. The issuer flow can be found in Issuer-credential-portal: https://github.com/Anushka3174/Issuer-credential-portal.git, the holder wallet is available in Holder-portal: https://github.com/Anushka3174/Holder-Portal.git, and the verifier under the repository named Bank-as-Web-Portal-Verifier: https://github.com/Anushka3174/Bank-as-Web-Portal-Verifier.git. A well-documented README.md file can be referred to for running the program on a local system.

## 7. Evaluation and Discussion

As a first step, we carefully selected experts who had long dealt with SSI or identity verification solutions during their daily work and could therefore evaluate our artifact. The experts were from different domains and companies related to our research including Signicat [63], the Decentralized Identity Foundation [69], Affinidi [55], and Decentralized Systems Lab (NTNU) [65]. Throughout the design and development process we conducted discussions with experts to evaluate our design and PoC implementation. Based on their feedback and the lessons from the PoC, we adapted our artifact. For instance, we added the selective disclosure feature that enabled users to share only the required attributes when presenting their credentials to a verifier. Moreover, based on the recommendation of the experts, it was decided to reuse existing credential schemas instead of creating a new one. That way our system would be more interoperable with other systems. We now consolidate our findings by providing a summary analysis, assessing the specified design objectives’ fulfillment.

- **Privacy, user control, and consent**. Our artifact supports verifiable credentials with a selective disclosure feature. Users can share only the required attributes in a document and hide optional ones, which minimizes data and enhances user control and privacy. Moreover, users manage their data independently with their identity wallet, giving them full control over their digital identities. Users get an overview in their digital wallet of which data they have shared and with whom. Furthermore, no personal data are stored at other parties or on a blockchain. Thus, compliance with the GDPR’s fundamental objectives can likely be achieved. In addition, by implementing our approach, user interactions are not trackable by issuing organizations because a verifier does not have to directly contact an issuer during the verification process.
- **Decentralization**. In our solution, users use a personal digital wallet to manage their keys and data, which means that unlike typical systems that store data centrally, credentials would be saved in digital wallets that would be distributed all over the edges of the network giving individual users full control over their personal data. This would also vastly increase the complexity of any kind of attack, and even if certain systems were penetrated, it would no longer be a massive honeypot containing millions of individuals’ personal information. It is undeniable that the existing standards and technologies have their limits. Equifax [70], Cambridge Analytica [71], and First American Financial [72] are the most recent examples of data breaches in which the identity information of millions of people was exposed. In addition, the use of distributed ledger technology as a verifiable data registry improves data reconciliation. Each time the user data are transformed, it opens up opportunities for data loss or incorrect data to enter the workstream. By having a decentralized data store, every entity has access to a real-time, shared view of the data. However, great consideration should be given to wallet and agent security, due to their critical role in holding and processing client identity information.
- **Authenticity and integrity**. Verifiable credentials play a major role in building the strength of the proposed system in terms of authenticity and integrity. Documents issued as VCs such as in our artifact are tamper-proof and authentic through the issuer’s digital signature. Any issuer-signed verifiable credential can be cryptographically checked, and information about who issued it and if it has been tampered with can be found in real time. Easy-counterfeit identification would help businesses find genuine applicants with authentic information about them and could turn away possible fraudulent users.
- **Transparency**. The decentralized method of online loan processing and document verification also makes the process transparent. It is important that the system used to administer and operate the process be open in regard to how it functions and how it is managed. Since the implemented system is based on open-source protocols and development tools, it provides more flexibility to examine how it works and if there are loopholes that one must be aware of. Further, it allows anyone to examine how different components operate, making the overall system transparent and trustworthy.
- **Interoperability**. The existing methods of document verification are mostly done through collaborative networks, but the process is not ideal in a sense that these require a preconnection with one another to be able to be fully functional. This process is expensive to service users and adds an overhead of financial burden instead of focusing on their core business idea. Similarly, a solution cannot be called interoperable if it only works for a certain collaborative network. In existing systems, there is no common methodology for validating and verifying electronic documents acceptable across multiple domains, as a result, systems are either siloed, fragmented, or limited to collaboration networks [3,73]. SSI on the other hand, is based on open standards and protocols and holds the possibility to be interoperable. Interoperability in SSI means that the credential should be used as widely as possible. The majority of SSI solution creators already base their work on two open W3C standards. As our solution is based on these open-standard specifications and vendor-neutral technology components, it maximizes interoperability and transitive trust while minimizing the possibility of vendor lock-in [5]. Moreover, the proposed process includes putting schema definitions based on JSON-LD on a public blockchain that all verifiers can access and examine to determine semantic interoperability. The capacity of computer systems to exchange data with a clear, shared meaning is known as semantic interoperability. In addition to data packaging (syntax), semantic interoperability is concerned with the simultaneous transmission of meaning and data (semantics). This is done by including metadata, which link each data element to a controlled, common vocabulary.
  One most notable feature in the proposed system is its integration with a trusted electronic authentication system such as BankID. Creating a verifiable credential based on BankID would bootstrap the initial trust and make the system interoperable with the existing traditional solutions. Financial organizations can verify any documents issued by various organizations easily in a secure way as long as they trust the issuer of the organization. This enables any credential to be used as widely as possible and across multiple domains while being completely secure and user-centric.
- **Efficiency**. Compared to existing methods, the potential efficiencies of the proposed method of online loan processing and document verification is far-ranging. There is usually a certain waiting time involved in verifying the authenticity of documents. This is because the verification is either done manually, through collaborative networks, or by verifying documents against different registries that may take a few days to multiple weeks, since financial institutions are heavy with regulations and this process is critical. Moreover, the verifier has to have some kind of connection with the issuer, i.e., when a user presents their documents to a verifier, it connects to the issuer through an API call or by using other ways of communication and then returns the response. This requires a handshake at different levels, taking more time and resources for the verifier.
  On the other hand, authenticity in the proposed method can be digitally verified in seconds, taking business efficiency to a new level. As credentials are cryptographically verifiable in real time, this would also improve user experience. In other words, it reduces the friction between the organization and the customer. In addition, in the proposed system, unlike collaborative networks, different levels of handshakes are limited to two levels, i.e., between holder and verifier, significantly improving the time for processing an application. Further, reducing the number intermediaries also reduces costs as financial institutions do not need to pay a third party for the verification services.

Overall, the proposed approach improves various aspects of the existing online processing and document verification system making it more efficient, reliable, interoperable, and privacy-preserving. The proposed system can impact online loan verification and processing in several ways. A customer can selectively disclose attributes from their credentials instead of the common practice of submitting all credentials, preserving privacy while also reducing a financial organization’s liability for holding data that they do not require. Another benefit is that the user uses digital wallets to manage their keys and data unlike typical systems that store data centrally. This gives users full control over their personal data while avoiding the creation of a centralized data store that could be a honeypot for hackers. The proposed system also provides transparency, authentication, and integrity. Tamper-evident verifiable credentials build the strength of the system by using cryptography and standard internet protocols, where the validity of a document can be verified in seconds. It also reduces handshakes at different levels and saves cost and time that would otherwise be required by using third-party services for document verification. This reduces the friction between customer and financial services and enhances faster services. It is important to note that the proposed system is based on open standards and protocols such as VC, DID, JSON-LD, thus, it is a vendor-neutral technology and provides maximum interoperability and a possibility for the credential to be used as widely as possible.

Through conversations with experts and on the basis of a detailed study about schemas and schema definitions, it was found there were no organizations in the SSI realm that specified the structure or specification of a credential definition for a certain use case. However, it is beneficial in a manner, as standard bodies produce enormous bureaucracy that slows growth and takes forever to be established. The credential definition should instead evolve organically and most likely be established through partnership between a few forward-thinking organizations who would agree on a credential definition and standards for what is required. At this point, a verifiable credential context and schema definitions are hypothetical, experimental, and under development.

The use of the distributed ledger technology for storing public keys of credential issuers and revocation registries for VCs provides an infrastructure that allows a bank to verify VCs issued by other banks or government institutions. Nonetheless, governance mechanisms regarding the legal acceptance of such VCs and other aspects of interbank collaboration requirements still leave some questions open. A transparent governance framework is necessary to clarify which credentials the banks accept and whom they accept as a credential issuer.

## 8. Conclusions

While several papers and projects have explored the use of blockchain for online document verification systems, none have focused on the integration of SSI technology to solve existing problems such as interoperability, lack of privacy, and user control. To address this research gap, we followed a DSR approach and by focusing on decentralized technologies, we proposed a novel framework for online document verification. By implementing and evaluating a PoC with the help of experts, we also demonstrated the feasibility of the SSI-based document verification approach and its fitness to solve current problems. We found that SSI allowed for reliable and efficient credential verification. Issuing certificates as VCs and using SSI’s privacy-oriented selective disclosure capabilities for online verification has considerable advantages for all users. By providing novel design principles, we uncovered valuable insights for SSI-based solutions in the context of online document verification.

Our research has limitations, which can stimulate further research. By incorporating well-established research methodology and getting input from experts, we refrained from testing with end users. However, we acknowledge that generic usability studies of the proposed system are an interesting future research endeavor. Moreover, there are further conceptual challenges to be solved before SSI is used in real systems and settings, especially regarding the necessary governance frameworks and a more detailed regulatory analysis. In particular, details regarding the cooperation of entities in the document verification process, the creation of trust registries, and the responsible parties for operating the blockchain when using a permissioned network must be clarified. This opens various promising avenues for future research. 

## Figures and Tables

**Table 1:** Comparison of solutions for document verification.

	DocBlock	IDStack	Marella and Vijayan	Schlatt et al.	Soltani et al.
**Year**	2021	2017	2020	2022	2018
**Use case**	Document verification	Document verification	Document verification	eKYC	eKYC
**Aligns with** **SSI standards**	No	No	No	Yes	Yes
**User control**	No	No	No	Yes	Yes
**User authentication**	Yes	No	No	Yes	Yes
**Validation (authenticity** **and integrity)**	SHA-256 hashing, ECDSA signatures	Text extraction, self-signed certificates	SHA-256 hashing, digital signatures	Cryptographic proofs in the form of VCs	Secure DID connections and VCs
**Verification process**	Automated	Manual	Automated	Automated	Automated
**Use of blockchain**	Yes (Ethereum)	No	Yes (Hyperledger Fabric)	Yes	Yes (Hyperledger Indy)
**Data storage**	IPFS	Local database (SQL)	Blockchain (hashes), local database (NoSQL)	Mobile and cloud wallets	Mobile and cloud wallets
**Technologies**	Smart contracts, Metamask wallet	APIs, JSON documents	Smart contracts, APIs	DIDs, VCs	DIDs, VCs
**Proof of concept**	Yes	Yes	Yes	No	Yes

**Table 2:** Comparison of different SSI frameworks.

	Mattr	Trinsic	Affinidi	Veramo
**Well-documented APIs**	Yes (website and Postman)	Yes (website)	Yes (website and Postman)	No (hard to follow)
**Developer Support**	Yes (Slack)	Good (chatbot and Slack)	Yes (Discord)	Yes (GitHub)
**Aligns with SSI standards**	Yes	Yes	Yes	Yes
**Availability of fully** **functional demo app**	No	Yes	Yes	No
**Regularly maintained**	Yes	Yes	Yes	Yes
**Supported DID**	did-key, did:ion, did:web	did:sov, did:peer, did:key, did:web	did-elem, did:jolo	did:ethr, did:web, did:key
**Distributed Ledgers**	Bitcoin	Sovrin	Etherium	Etherium

## Data Availability

Not Applicable.
